# Identification of target genes of PAX3-FOXO1 in alveolar rhabdomyosarcoma

**DOI:** 10.3892/or.2013.2513

**Published:** 2013-06-03

**Authors:** EUN HYUN AHN, GABRIELA E. MERCADO, MARICK LAÉ, MARC LADANYI

**Affiliations:** 1 Department of Pathology and Laboratory Medicine, University of Pennsylvania School of Medicine, Philadelphia, PA 19104, USA; 2 Department of Pathology, University of Washington School of Medicine, Seattle, WA 98195, USA; 3 Department of Pathology, Memorial Sloan-Kettering Cancer Center, New York, NY 10021, USA; 4 Human Oncology and Pathogenesis Program, Memorial Sloan-Kettering Cancer Center, New York, NY 10021, USA

**Keywords:** rabdomyosarcoma, PAX3-FOXO1, gene expression, microarray

## Abstract

Rhabdomyosarcoma (RMS) is a soft tissue sarcoma categorized into two major subtypes: alveolar RMS (ARMS) and embryonal RMS (ERMS). Most ARMS express the PAX3-FOXO1 (P3F) fusion oncoprotein generated by the 2;13 chromosomal translocation. In the present study, the downstream target genes of P3F were identified by analyzing two independent sets of gene expression profiles: primary RMS tumors and RD ERMS cells transduced with inducible P3F constructs. We found 34 potential target genes (27 upregulated and 7 downregulated) that were significantly and differentially expressed between P3F-positive and P3F-negative categories, both in primary RMS tumors and in the inducible P3F cell culture system. Gene ontology analysis of microarray data of the inducible P3F cell culture system employed indicated apoptosis, cell death, development, and signal transduction as overrepresented significant functional categories found in both upregulated and downregulated genes. Therefore, among the 34 potential target genes, the expression of cell death-related [Gremlin1, cysteine knot superfamily 1, BMP antagonist 1 (GREM1) and death-associated protein kinase 1 (DAPK1)] and development-related [myogenic differentiation 1 (MYOD1) and hairy/enhancer-of-split related with YRPW motif 1 (HEY1)] genes were further investigated. The differential expression of GREM1, DAPK1, MYOD1 and HEY1 was confirmed in independent tumors and inducible cell culture systems. The expression of GREM1, DAPK1 and MYOD1 were significantly upregulated; HEY1 was significantly downregulated in independent P3F-positive ARMS tumors and transcriptionally active P3F cells, compared to those in ERMS tumors and transcriptionally inactive P3F cells. This study identified target genes of P3F and suggested that four downstream targets (GREM1, DAPK1, MYOD1 and HEY1) can contribute to the biological activities of P3F involved in growth suppression or cell death and myogenic differentiation.

## Introduction

Rhabdomyosarcoma (RMS), the most common type of pediatric soft tissue sarcoma, is associated with the skeletal muscle lineage. Pediatric RMS is categorized into two major subtypes: embryonal RMS (ERMS) and alveolar RMS (ARMS) based on their histologic appearance (1). ERMS shows a more favorable prognosis, while ARMS is more aggressive with a high frequency of metastases at initial diagnosis and a worse prognosis (2,3).

ERMS has not been found to be associated with any diagnostic genetic alterations, but loss of heterozygosity at 11p15 is a common finding (4,5). In contrast, ARMS is associated with recurrent chromosomal translocations. The translocation t(2;13)(q35;q14) leading to the PAX3-FOXO1 (P3F) gene fusion was found to be present in 55% of ARMS cases, while the translocation t(1;13)(q36;q14) leading to the PAX7-FOXO1 gene fusion was present in 22% of cases, and 23% of ARMS were fusion-negative (2). ARMS with the 2;13 translocation is characterized as having overexpression of P3F relative to wild-type PAX3, at both the RNA and protein levels (6). In both PAX3 and P3F, an alternative splice occurs at the intron 2/exon 3 junction resulting in inclusion or exclusion of a glutamine (Q) residue [PAX3(+Q) or (−Q) forms, respectively] (7). As a result, PAX3(+Q)-FOXO1 and PAX3(−Q)-FOXO1 are coexpressed in P3F-positive ARMS tumors.

Among RMS tumors, PAX3(+/−Q)-FOXO1-positive ARMS is the most clinically intractable fusion subtype of pediatric RMS (1,2,8,9). However, the histologic classification of RMS into ARMS and ERMS may be difficult in some cases (9) and there are no specific drugs for treating specific histologic or fusion subtypes. Therefore, there is substantial impetus to elucidate target genes of P3F, which can be used to identify therapeutic targets and markers for use in RMS diagnosis and management.

Although several recent studies utilized gene expression profiles to classify RMS and/or identify target genes of P3F and PAX7-FOXO1 or P3F only in ARMS (10–16), more research is needed to validate the function of genes that are biologically relevant in ARMS development.

In the present study, we identified potential target genes of P3F by analyzing gene expression profiles from two independent systems: primary tumors (P3F-positive ARMS and fusion-negative ERMS) and a cell culture system expressing the inducible PAX3(+/−Q)-FOXO1-estrogen receptor (ER) ligand binding domain construct in the pBabe (pB) retroviral vector. Among the potential target genes of P3F, we focused on cell death or apoptosis-related [Gremlin1, cysteine knot superfamily 1, BMP antagonist 1 (GREM1), death-associated protein kinase 1 (DAPK1)] and development-related [myogenic differentiation 1 (MYOD1) and hairy/enhancer-of-split related with YRPW motif 1 (HEY1)] genes since apoptosis and development were significantly overrepresented functional categories in our gene expression profiles.

## Materials and methods

### Tumor samples

The tumor specimens (15 ERMS and 16 P3F-positive ARMS) used for microarray were previously described (15). Independent tumor specimens (20 ERMS and 17 P3F-positive ARMS) examined for quantitative reverse-transcription PCR (qRT-PCR) in the validation studies were previously described (15). The presence of P3F in ARMS tumor specimens was determined and confirmed by RT-PCR and/or qRT-PCR (17).

### Cell culture

RD ERMS cell line and RD-derived cells transduced with inducible PAX3(+/−Q)-FOXO1 in pB or pK1 (pK) were maintained in Dulbecco’s modified Eagle’s medium (DMEM) (Invitrogen, Grand Island, NY, USA) with 10% fetal bovine serum (FBS) (Thermo Scientific HyClone, Logan, UT, USA). The medium was supplemented with 1% penicillin/streptomycin (P/S) and 1% antibiotic-antimycotic (AM) (both from Invitrogen).

### DNA constructs and construction of PAX3(−Q)-FOXO1-ER in pB retroviral vector

Retroviral constructs with several relevant DNA inserts [pK1-PAX3(+Q)-FOXO1-ER (pK-P3F-ER), pBabe-PAX2(+Q)-FOXO1-ER (pB-P3F-ER) and pCDNA3-PAX3(+Q)-FOXO1-ER (pCDNA-P3F-ER)] were previously generated by Dr Frederic G. Barr’s laboratory (18–21). A modified ER ligand-binding domain was provided by Dr G.I. Evan (22). Unless it is noted as PAX3(−Q)-FOXO1, the P3F constructs used in our studies refer to the PAX3(+Q)-FOXO1 isoform. In order to clone an inducible construct of PAX3(−Q)-FOXO1-ER into the pB retroviral vector, two consecutive mutations were introduced, using QuikChange site-directed mutagenesis (Stratagene; Agilent Technologies, Santa Clara, CA, USA) followed by multiple subcloning steps. Protocols are available upon request.

### Establishment of ERMS cell culture systems inducibly expressing P3F

Retroviral transduction was performed as described previously with modifications (20,21). Cells that were transduced with retroviral DNA constructs were selected with puromycin (BD Biosciences, San Jose, CA, USA) at 1 μg/ml for RD-derived cells with PAX3(+/−Q)-FOXO1-ER in pB or in pK1. Cells carrying inducible PAX3(+/−Q)-FOXO1-ER were treated with the ligand 4-hydroxytamoxifen (Tmf) (Sigma-Aldrich, St. Louis, MO, USA), which activates and induces transcriptional activity of PAX3(+/−Q)-FOXO1-ER by translocating PAX3(+/−Q)-FOXO1-ER to the nucleus.

### Luciferase reporter assays

RD cells expressing PAX3(+/−Q)-FOXO1-ER in pB or pB alone were plated at 5×10^4^ per well in 24-well plates. The cells were transfected with 0.3 μg firefly luciferase reporter DNA containing PAX3 DNA binding sites (6 × PRS9) and 0.015 μg pRL-TK Renilla luciferase DNA using FuGENE^®^ 6 (both from Promega, Madison, WI, USA) and incubated for 2 days. The transcriptional activity of PAX3(+/−Q)-FOXO1 was measured by the dual-luciferase assay (Promega) and was normalized by transfection efficiency control (pRL-TK *Renilla* luciferase).

### RNA extraction and microarray data analysis

For microarray analysis, total RNA was extracted using the RNeasy Mini Kit (Qiagen, Valencia, CA, USA). For other studies including qRT-PCR and RT-PCR, total RNA was extracted using RNA STAT-60 (Tel-Test, Inc., Friendswood, TX, USA). Microarray analysis of RNA isolated from RMS tumors and RD cell culture systems expressing inducible PAX3(+/−Q)-FOXO1-ER in pB was performed on Affymetrix arrays. Microarray analysis of 31 RNA tumor samples (15 ERMS and 16 P3F-positive ARMS) was described in our previous publication (15).

For microarray analysis of inducible cell culture systems, three RD cell populations were independently transduced with each construct [PAX3(+Q)-FOXO1-ER in pB; PAX3(−Q)-FOXO1-ER in pB; pB vector alone] and treated without or with 30 nM Tmf for 24 h to generate a total of 18 samples. RNA isolated from these cells was hybridized onto Affymetrix GeneChip Human Genome U133 Plus v.2.0 (HG U133+ v.2.0). After the expression levels were determined using Affymetrix Microarray Suite 5.0, the expression levels were normalized in GeneSpring (Agilent Technology). The probe-sets, whose expression levels were detected in at least 2 of the 18 samples (in inducible cell culture system) or 1 of the 31 samples (in tumors), were selected for further analysis. To compare the gene expression profiles from tumors with those from the inducible cell culture systems, the probes on the HG U133A array were used for GeneSpring analysis.

The Significance Analysis of Microarrays (SAM) (23) with two-class paired condition was used as the common method to identify differentially expressed genes between 16 P3F positive-ARMS and 15 fusion-negative ERMS tumors as well as between the RD cells expressing PAX3(+/−Q)-FOXO1-ER in pB (treated with Tmf; 6 samples) and the RD cells expressing PAX3(+/−Q)-FOXO1-ER in pB (not treated with Tmf; 6 samples). Data was filtered by a false discovery rate (FDR) at <5% (FDR 4.94% for cells; FDR 4.91% for tumors) and ≥1.5 fold-change. The PAX3(+Q)-FOXO1-ER and PAX3(−Q)-FOXO1-ER gene expression profiles were pooled since SAM did not reveal significant differentially expressed genes between these two groups. The genes that were differentially expressed between transcriptionally active PAX3(+/−Q)-FOXO1-ER (Tmf-treated) and inactive PAX3(+/−Q)-FOXO1-ER (untreated control) were then examined.

Finally, common genes that were present in both tumors and the inducible PAX3(+/−Q)-FOXO1-ER cell culture system were identified by Venn Diagram analysis. After this analysis, the probe-sets that encoded the same gene with similar expression profiles and showed raw expression values at <150 were removed. Classifications and functional annotations of genes were analyzed via web-accessible programs: Expression Analysis Systematic Explorer (EASE) 2010 and Database for Annotation, Visualization and Integrated Discovery (DAVID) 2010 (24).

### Quantitative reverse transcription-PCR (qRT-PCR) analysis

The qRT-PCR assay was performed as described previously (15,16). Test gene assays were normalized to the expression of 18S rRNA. Taqman gene expression assays used (Applied Biosystems) were: *DAPK1* (assay ID# Hs00234489_m1), *GREM1* (assay ID# Hs00171951_m1) and *MYOD1* (assay ID# Hs00159528_m1). The sequences of forward and reverse primers and probes of PAX3-FOXO1 and HEY1 are available upon request.

### Extraction of cellular proteins and isolation of secreted proteins from the medium of cultured cells and immunoblot analysis

The cells were seeded at 10^6^/100-mm dish in phenol red-free DMEM medium containing 10% FBS, 1% P/S and 1% AM for 24 h prior to Tmf treatment. The cells expressing inducible PAX3(+/−Q)-FOXO1-ER were then treated with 30 nM Tmf for various times. Cellular proteins were lysed as described previously (20). To assay the secreted GREM1 protein, the medium containing 10% FBS was replaced with FBS-free DMEM after 24 h of Tmf treatment, and cells were cultured for an additional 24 or 48 h with Tmf treatment. The medium of the cultured cells was collected and concentrated to <1,000 μl using Amicon Ultra-4 with Ultracel-10K (UFC80-1024; Millipore, Billerica, MA, USA). Protein was quantified using Coomassie Plus Protein Assay (Pierce Biotechnology, Inc., Rockford, IL, USA).

Immunoblot analysis was performed as described previously (25) with modifications. The primary antibodies used were: anti-PAX3 rabbit polyclonal, DAPK1 (ab10443; both from Abcam, Cambridge, MA, USA) and GREM1 (cat# AP6133a; Abgent, San Diego, CA, USA).

### Statistical analysis

The qRT-PCR data of tumors (20 fusion-negative ERMS and 17 P3(+Q)F-positive ARMS) were analyzed by the Mann-Whitney Test (Wilcoxon Rank Sum Test) and differences between the two tumor groups were considered significant at a P-value <0.05. Statistical analysis of gene expression profiles are described in ‘RNA extraction and microarray data analysis’.

## Results

### Development of inducible PAX3(+/−Q)-FOXO1 cell culture systems

**Figure 1:**
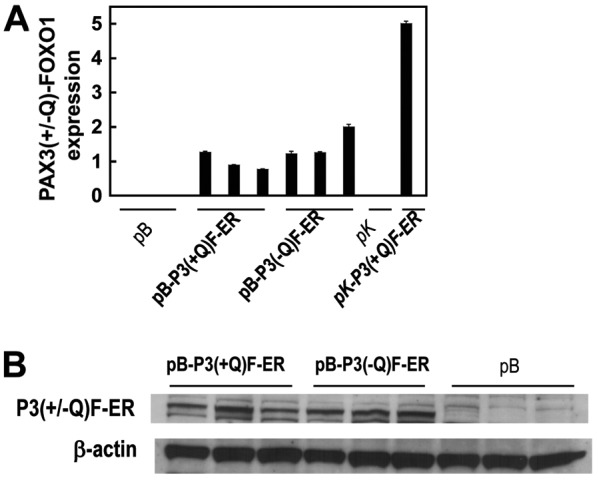
Expression of inducible forms of PAX3(+/−Q)-FOXO1 in RD cells transduced with pBabe (pB), pB-PAX3(+Q)FKHR-ER [pB-P3(+Q)F-ER], pB-PAX3(−Q)FKHR-ER [pB-P3(−Q)F-ER], pK1 (pK), or pK-PAX3(+Q)-FOXO1-ER [pK-P3(+Q)F-ER]. (A) Total RNA was isolated and the expression of PAX3(+/−Q)-FOXO1 was determined by qRT-PCR and was normalized to 18S rRNA expression (means ± SD). (B) Total protein was extracted after 2 weeks of puromycin selection from RD ERMS cells transduced with pB-P3(+Q)F-ER (lanes 1–3), pB-P3(−Q)F-ER (lanes 4–6), pB vector alone (lanes 7–9).

RD ERMS cell line was transduced with PAX3(+/−Q)-FOXO1-ER in pB [pB-P3(+/−Q)F-ER] or pB alone and selected with puromycin for 2 weeks. The transduction of RD cells with these pB-P3(+/−Q)F-ER constructs resulted in the expression of PAX3(+/−Q)-FOXO1-ER at both the mRNA (Fig. 1A) and protein levels (Fig. 1B).

**Figure 2:**
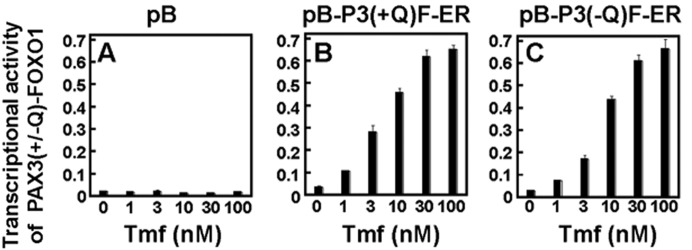
Transcriptional activity of inducible forms of PAX3-FOXO1 (P3F) in RD cells transduced with (A) pBabe (pB), (B) pB-PAX3(+Q)FKHR-ER [pB-P3(+Q)F-ER], or (C) pB-PAX3(−Q)FKHR-ER [pB-P3(−Q)F-ER]. The cells were seeded at 5×10^4^ cells/well, and transcriptional activity was determined by dual luciferase assay with a reporter containing PAX3 DNA binding sites (6 × PRS9) and then the cells were treated with tamoxifen at 1, 3, 10, 30 and 100 nM for 24 h. The luciferase activity was normalized by transfection efficiency control (pRL-TK-*Renilla*). Data shown are means ± SEM (n=3). Where an error bar is not shown, it lies within the dimensions of the symbol.

In order to determine and confirm the inducible PAX3(+/−Q)-FOXO1 function, the RD-derived cells transduced with pB-P3(+/−Q)F-ER or pB were transiently transfected with a luciferase gene reporter construct containing PAX3 DNA binding sites and were treated with tamoxifen at various concentrations (0, 1, 3, 10, 30 and 100 nM) for 24 h (Fig. 2). The RD cells transduced with an empty vector pB alone (Fig. 2A) showed a very low (~0.02) transcriptional activity indicating only an endogenous PAX3 transcriptional activity. In contrast, RD cells transduced with pB-P3(+Q)F-ER (Fig. 2B) or pB-P3(−Q)F-ER (Fig. 2C) demonstrated much higher transcriptional activities as Tmf concentrations increased. The maximal transcriptional activity in RD-derived cells with pB-P3(+/−Q)F-ER was observed at 30 nM Tmf and showed a ~46- to 47-fold difference in luciferase gene transactivation between RD-P3(+/−Q)F-ER cells and RD-pB cells (Fig. 2B and C).

### Microarray data analysis of tumors and inducible cell culture system identifies 34 potential target genes of P3F

To identify target genes of P3F in the present study, we utilized RD cells expressing inducible PAX3(+/−Q)-FOXO1-ER constructs in the pB vector (Figs. 1 and 2) based on the following rationale and significance. First, because the expression of PAX3(+/−Q)-FOXO1 in ARMS tumors varies, the utilization of cell culture models having various levels of PAX3(+/−Q)-FOXO1 can provide an unbiased approach for identifying potential target genes of PAX3(+/−Q)-FOXO1. Our previous gene expression profiling study investigated RD cells transduced with an inducible PAX3(+Q)-FOXO1-ER construct in the pK vector that enforces high expression of PAX3(+Q)-FOXO1-ER (16). In contrast, our present expression profiling study used the pB vector to express lower levels of inducible PAX3(+/−Q)-FOXO1-ER in RD cells. Second, a previous expression profiling analysis of the inducible cell system (16) was complicated by the confounding factor of a treatment-day effect. Thus, microarray data from inducible cells with PAX3(+Q)-FOXO1-ER in pK were analyzed by Mixed ANOVA, whereas primary RMS tumors were analyzed by SAM. In comparison, no such confounding factors were present in the present study and thus microarray data from both primary RMS tumors and inducible cells were both analyzed by SAM.

After analyzing microarray data from primary RMS tumors as well as inducible cells [PAX3(+/−Q)-FOXO1-ER in pB] by SAM, data were filtered for expression values, FDR, and fold-change (FDR at <5% and ≥1.5 fold-change). In the tumor microarray data, 1,693 probe-sets (1,115 upregulated and 578 downregulated) on the HG U133A platform were either significantly upregulated or downregulated in the PAX3(+/−Q)-FOXO1-positive ARMS tumors (n=16) compared to the fusion-negative ERMS tumors (n=15). Analysis of genes differentially expressed between induced (transcriptionally active) PAX3(+/−Q)-FOXO1-ER in pB (Tmf treated, n=6) and uninduced PAX3(+/−Q)-FOXO1-ER in pB (untreated control, n=6) revealed 540 probe sets (304 upregulated and 236 downregulated) on the HG U133+ v.2.0 array. Of these 540 probe sets, 328 were present on the HGU133A array (182 upregulated and 146 downregulated). Finally, 34 potential target genes (27 upregulated and 7 downregulated) were differentially expressed both in tumors and in the inducible cell culture system, using probes from the HG U133A array (Table I).

### Many of the differentially expressed genes in the PAX3(+/−Q)-FOXO1-ER cell systems are related to apoptosis and development

**Figure 3:**
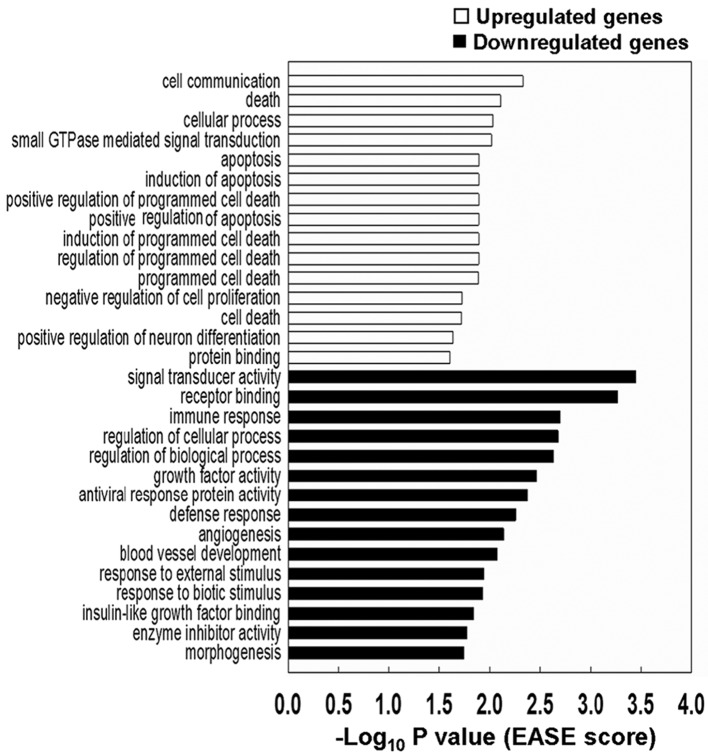
Expression analysis systematic explorer (EASE) of gene ontology annotations in gene expression profiles of the inducible PAX3-FOXO1 (P3F) cell systems. Functional categories enriched in the significantly upregulated (□) and downregulated (■) genes in the gene expression profiles of inducible P3F cell systems are shown.

To investigate biological consequences of the PAX3(+/−Q)-FOXO1 expression signature, the 540 significantly upregulated or downregulated probes identified in RD cells expressing the inducible PAX3(+/−Q)-FOXO1-ER in pB on the HG-U133+ v.2.0 platform were analyzed using EASE and DAVID (24). Notably, 10 out of the 15 most significant functional categories analyzed by EASE for the upregulated genes were apoptosis, cell death, or negative regulation of cell proliferation (Fig. 3).

The most significant 20 overrepresented functional groups in the RD cells carrying inducible PAX3(+/−Q)-FOXO1-ER in pB identified by DAVID are listed in Table II. Among these 20 groups, five groups were commonly found in both upregulated and downregulated genes. Four out of these 5 functional groups were related to development (developmental process, multicellular organismal development, anatomical structure development, system development) and one group to signal transduction. *MYOD1* (an upregulated gene) and *HEY1* (a downregulated gene) were found in all of the 5 common functional groups.

### GREM1, DAPK1, MYOD1 and HEY1 are significantly and differentially expressed in both independent tumors and inducible PAX3-FOXO1-ER cell systems

**Figure 4:**
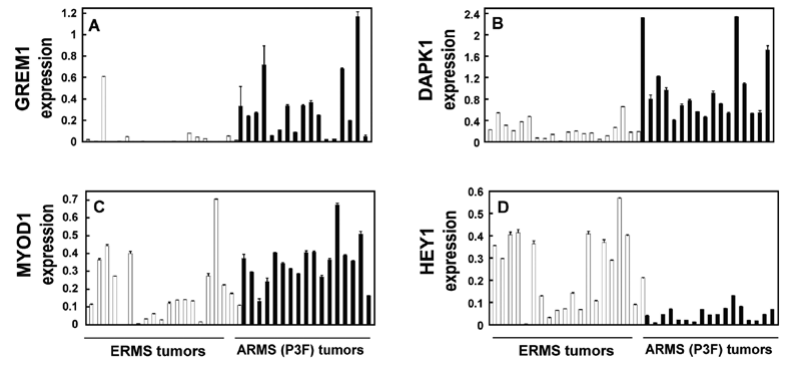
Validation of expression of genes in an independent set of tumors using qRT-PCR. The expression of GREM1, DAPK1, MYOD1 and HEY1 was determined by qRT-PCR in fusion-negative ERMS (n=20, □) and PAX3-FOXO1 (P3F)-positive ARMS (n=17, ■) primary tumors. Total RNA was isolated and the expression levels of these genes were normalized to 18S rRNA expression (mean ± SD). The mean expression for the 4 genes in P3F-positive ARMS were significantly different (P<0.005; see Table I) from those of the ERMS, respectively.

**Figure 5:**
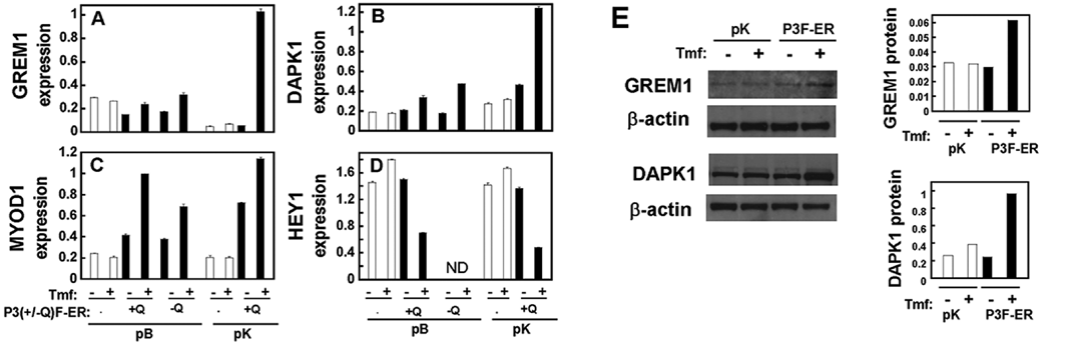
Validation of the expression of genes in the RD cells transduced with PAX3(+/−Q)-FOXO1-ER using qRT-PCR and immunoblot assays. (A-D) RD cells transduced with +Q or −Q isoforms of PAX3(+/Q)-FOXO1-ER [P3(+/−Q)F-ER] (■) in pBabe (pB) or P3(+Q)F-ER in pK1 (pK) (■) or retroviral vectors (□) alone (pB or pK), were treated without or with 4-hydroxytamoxifen (Tmf) at 30 nM for 24 h. Total RNA was isolated and the expression of these genes was determined by qRT-PCR. The relative expression levels of the 4 genes were normalized to 18S rRNA (mean ± SD; n=3). (E) Cells were cultured for 48 h with 30 nM Tmf. Total proteins were extracted and protein expression was determined by immunoblot analysis. The protein expression levels of GREM1 and DAPK1 were normalized by β-actin protein expression for densitometry graphs. ND, not determined.

Since gene ontology analysis EASE or DAVID indicated apoptosis (Fig. 3) and development (Table II) as significant functional categories, we validated the expression of apoptosis-related genes (*GREM1, DAPK1*) and development-related genes (*MYOD1, HEY1*) that were identified among the 34 potential target genes (Table I) differentially expressed in both primary tumors and the inducible cell culture system. These genes were analyzed in an independent panel of primary tumors (20 fusion-negative ERMS and 17 PAX3-FOXO1-positive ARMS) (Fig. 4; Table III) and RD cells carrying inducible PAX3(+/−Q)-FOXO1-ER in pB or pK (Tmf-treated versus untreated control) (Fig. 5; Table III). The validation was carried out at the RNA level using qRT-PCR for the expression of *GREM1* (Figs. 4A and 5A), *DAPK1* (Figs. 4B and 5B), *MYOD1* (Figs. 4C and 5C) and *HEY1* (Figs. 4D and 5D). Furthermore, the upregulation of GREM1 and DAPK1 at the protein level was validated using immunoblot analysis (Fig. 5E). These validation studies of GREM1, DAPK1, MYOD1 and HEY1 (Figs. 4 and 5) consistently demonstrated significant differential expression, which was comparable to that noted in the microarray expression signature (Table III).

## Discussion

In the present study, we developed the RD ERMS cell culture system expressing the inducible PAX3(+/−Q)-FOXO1-ER in pB, which is useful for determining the early short-term effects of PAX3(+/−Q)-FOXO1 and for regulating the strength and duration of transcriptional activity of PAX3-FOXO1. We analyzed two independent sets of gene expression profiles: primary RMS tumors and RD ERMS cells transduced with inducible PAX3(+/−Q)-FOXO1 constructs. We found 34 potential target genes (27 upregulated and 7 downregulated) that were significantly and differentially expressed between PAX3(+/−Q)-FOXO1-positive and fusion-negative categories, in both primary tumors and the inducible PAX3(+/−Q)-FOXO1 cell culture system. Among the 34 genes, we investigated cell death or apoptosis-related (*GREM1*, *DAPK1*) and development-related genes (*MYOD1*, *HEY1*).

Target genes of PAX3-FOXO1 and PAX7-FOXO1, or PAX3-FOXO1 only, were previously reported, based on gene expression profiling in both primary tumors and cells transduced with constitutive PAX3/PAX7-FOXO1 (13) or inducible PAX3-FOXO1-ER (16) constructs. Constitutive PAX3-FOXO1 cell system represents relatively longer (later) effects of PAX3-FOXO1 compared to the inducible PAX3-FOXO1-ER cell systems. These two studies (13,16) reported 81 and 39 target genes, respectively. Compared with these two previous studies, 11 and 12 target genes, respectively, were found in common with the present study (Table IV). Six genes (*KCNN3, MCAM, MYOD1, TCF7L2, TM4SF10, ZFP36L2*) were identified as targets in all three studies (Table IV) (13,16, present study).

We demonstrated that higher expression of *MYOD1* and lower expression of *HEY1* were consistently observed in all PAX3-FOXO1-expressing ARMS primary tumors and cells, in comparison to fusion-negative ERMS primary tumors and cells. These findings of *MYOD1* upregulation are in accord with results reported in previous studies of MYOD1 expression in human ARMS (26,27), PAX3/PAX7-FOXO1-positive cells (13), mesenchymal stem cells transfected with PAX3-FOXO1 (28), and NIH3T3 fibroblasts transduced with PAX3-FOXO1 (10,29). In a previous study (30), HEY1 overexpression was found to inhibit MYOD1, an early myogenic differentiation marker, which indicates that HEY1 downregulation found in our study is consistent with this PAX3-FOXO1-induced myogenic developmental program including MYOD1 upregulation. It is hypothesized that PAX3-FOXO1 simultaneously reinforces myogenic determination by upregulating MYOD1, while suppressing terminal myogenic differentiation (31,32).

The oncogenic activity of PAX3-FOXO1 is characterized in part by its stimulatory effects on cell proliferation (33–35) and cell survival/anti-apoptosis (36,37) as well as its inhibitory role on terminal myogenic differentiation (31,32). However, in addition to these effects, PAX3-FOXO1 can also cause growth suppression and cell death in other settings (20,21,38). These paradoxical features of PAX3-FOXO1 being both oncogenic and growth-suppressive were demonstrated in previous studies with immortalized murine fibroblasts (20,21) and human myoblasts (38). We identified and validated that tumor-suppressor genes, GREM1 (39) and DAPK1 (40), are upregulated at both RNA and protein levels in PAX3-FOXO1-positive ARMS tumors and PAX3-FOXO1-ER inducible cell culture systems. Our study suggests that GREM1 and DAPK1 tumor suppressor genes can be potential target genes contributing to this growth-suppressive activity of high PAX3-FOXO1 expression.

**Figure 6:**
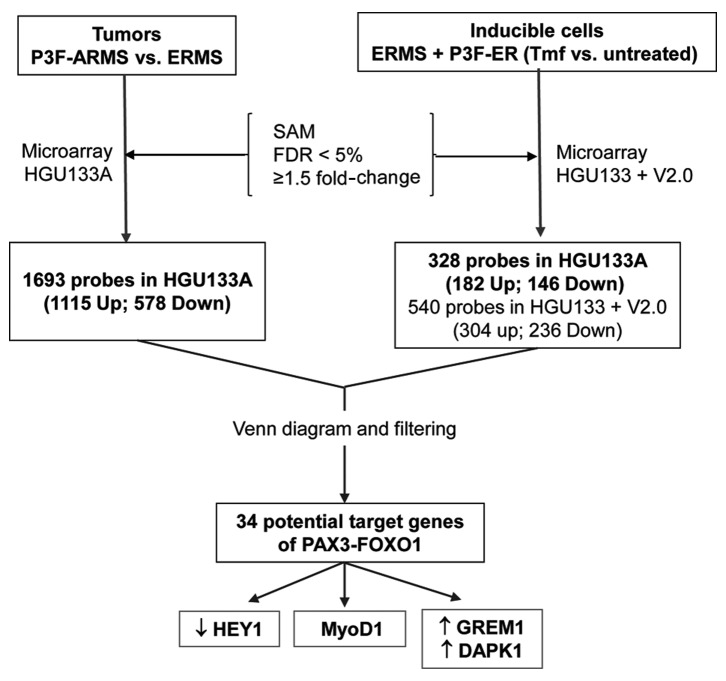
Identification of GREM1, DAPK1, and MYOD1 and HEY1 as potential target genes of PAX3-FOXO1 (P3F) in alveolar rhabdomyosarcoma (ARMS) based on two independent sets of gene expression profiles: primary RMS tumors and RD ERMS cells transduced with inducible P3F constructs.

In conclusion, we identified 34 potential downstream target genes of PAX3(+/−Q)-FOXO1 by analyzing two independent sets of gene expression profiles: primary RMS tumors and RD ERMS cells transduced with inducible PAX3-FOXO1 constructs. Our study can serve as a basis to propose the 4 genes (*GREM1, DAPK1, MYOD1* and *HEY1*) as targets that function in growth suppression or myogenic differentiation downstream of PAX3-FOXO1 in ARMS (Fig. 6).

## Figures and Tables

**Table I:** Potential target genes of PAX3-FOXO1 as identified by microarray analysis.

			Fold-change	
			
Affymetrix ID	Symbol	Gene name	Tumors (n=31)	Inducible cells (n=12)
Downregulated genes in P3F-positive categories of tumors and cells				
218974_at	FLJ10159	Hypothetical protein FLJ10159	2.85	1.82
**218839_at**	**HEY1**	**Hairy/enhancer-of-split related with YRPW motif 1**	**6.21**	**1.72**
209030_s_at	IGSF4	Cell adhesion molecule 1 (Immunoglobulin superfamily, member 4)	2.86	1.53
203708_at	PDE4B	Phosphodiesterase 4B, cAMP-specific (phosphodiesterase E4 dunce homolog, *Drosophila*)	4.80	3.77
202732_at	PKIG	Protein kinase (cAMP-dependent, catalytic) inhibitor γ	1.85	1.69
209875_s_at	SPP1	Secreted phosphoprotein 1 (osteopontin, bone sialoprotein I, early T-lymphocyte activation 1)	6.43	2.14
201368_at	ZFP36L2	Human Tis11d gene, complete cds.	2.60	1.56
Upregulated genes in P3F-positive categories of tumors and cells				
209459_s_at	ABAT	4-Aminobutyrate aminotransferase	10.52	5.84
206704_at	CLCN5	Chloride channel 5 (nephrolithiasis 2, X-linked, Dent disease)	2.68	2.01
**203139_at**	**DAPK1**	**Death-associated protein kinase 1**	**2.51**	**2.40**
213712_at	ELOVL2	Catenin (cadherin-associated protein), α-like 1	4.16	1.92
**218469_at**	**GREM1**	**Gremlin1, cysteine knot superfamily 1, BMP antagonist 1**	**5.34**	**2.13**
203233_at	IL4R	Interleukin 4 receptor	2.03	1.74
203126_at	IMPA2	Inositol(myo)-1(or 4)-monophosphatase 2	2.90	1.92
202794_at	INPP1	Inositol polyphosphate-1-phosphatase	1.89	1.51
205902_at	KCNN3	Potassium intermediate/small conductance calcium-activated channel, subfamily N, member 3	6.40	1.97
204094_s_at	KIAA0669	TSC22D2, TSC22 domain family, member 2	4.63	2.18
218829_s_at	KIAA1416	CHD7, chromodomain helicase DNA binding protein 7	2.26	2.31
211042_x_at	MCAM	Melanoma cell adhesion molecule	2.14	1.73
213256_at	MGC48332	Hypothetical protein MGC48332	4.35	1.52
**206657_s_at**	**MYOD1**	**Myogenic differentiation 1**	**2.59**	**2.28**
209106_at	NCOA1	Nuclear receptor coactivator 1	3.15	1.66
209289_at	NFIB	Nuclear factor I/B	1.97	1.51
205858_at	NGFR	Nerve growth factor receptor (TNFR superfamily, member 16)	4.03	1.77
204105_s_at	NRCAM	Neuronal cell adhesion molecule	6.62	2.36
209123_at	QDPR	Quinoid dihydropteridine reductase	5.04	1.84
203217_s_at	SIAT9	Sialyltransferase 9 (CMP-NeuAc:lactosylceramide α-2,3-sialyltransferase; GM3 synthase)	2.43	1.84
203625_x_at	SKP2	S-phase kinase-associated protein 2 (p45)	2.64	1.91
213624_at	SMPDL3A	Sphingomyelin phosphodiesterase, acid-like 3A	2.05	1.76
212761_at	TCF7L2	Transcription factor 7-like 2 (T-cell specific, HMG-box)	2.68	1.59
209656_s_at	TM4SF10	Transmembrane 4 superfamily member 10	2.36	3.11
215389_s_at	TNNT2	Troponin T2, cardiac	5.39	1.86
219038_at	ZCWCC2	Zinc finger, CW-type with coiled-coil domain 2	3.52	2.02
49111_at	DKFZp762M127	MRNA; cDNA DKFZp762M127 (from clone DKFZp762M127)	6.60	8.23

The 34 genes were significantly differentially expressed between P3F positive-ARMS and fusion-negative ERMS tumors and between induced (transcriptionally active) PAX3(+/−Q)-FOXO1-ER in pB and uninduced (inactive) PAX3(+/−Q)-FOXO1-ER in pB in RD-derived cells. The fold-change difference was calculated by dividing the expression levels of P3F-positive categories of tumors (ARMS) and inducible cells with those of P3F-negative categories of tumors (ERMS) and inducible cells. P3F, PAX3-FOXO1; ARMS, alveolar rhabdomyosarcoma; ERMS, embryonal rhabdomyosarcoma; pB, pBabe.

**Table II:** DAVID analysis of the microarray data of the inducible PAX3-FOXO1 cell culture system.

Gene category	Counts (no. of genes)	No. of probes	P-value
Upregulated genes in transcriptionally active PAX3(+/−Q)-FOXO1 cells			
Protein binding	112	163	1.08E-08
**Developmental process**	62	91	3.04E-06
**Multicellular organismal development**	44	63	2.60E-04
Ras GTPase binding	6	7	4.32E-04
Enzyme binding	11	19	5.41E-04
**Anatomical structure development**	40	65	6.75E-04
Small GTPase binding	6	7	7.56E-04
GTPase binding	6	7	1.24E-03
Regulation of cellular process	67	96	1.48E-03
Binding	151	209	1.49E-03
Cell communication	63	94	1.88E-03
**Central nervous system development**	10	14	2.09E-03
**System development**	33	50	2.13E-03
Splice variant	62	96	3.27E-03
Glycolipid metabolic process	4	5	3.68E-03
Regulation of biological process	69	99	3.94E-03
Transcription factor binding	12	19	4.11E-03
**Signal transduction**	57	84	4.36E-03
Apoptosis	10	12	4.43E-03
Transcription cofactor activity	10	14	4.88E-03
Downregulated genes in transcriptionally active PAX3(+/−Q)-FOXO1 cells			
Immune system process	26	36	5.41E-05
Glycoprotein	57	77	5.48E-05
Anatomical structure morphogenesis	25	35	9.55E-05
**Developmental process**	50	67	1.39E-04
**Anatomical structure development**	37	50	1.97E-04
**Multicellular organismal development**	39	53	2.46E-04
Response to virus	7	11	2.99E-04
von Willebrand factor, type C	5	8	3.21E-04
Immune response	21	31	3.59E-04
2-5-Oligoadenylate synthetase	3	5	4.45E-04
Negative regulation of biological process	24	35	4.58E-04
Signal	44	60	5.68E-04
**Signal transduction**	53	68	6.54E-04
2-5-Oligoadenylate synthetase, ubiquitin like region	3	5	7.37E-04
VWC (von Willebrand factor (vWF) type C domain)	5	8	8.24E-04
Response to external stimulus	16	23	8.50E-04
PIRSF005680:Interferon-induced 56K protein	3	5	9.61E-04
Morphogenesis of an epithelium	6	6	1.05E-03
**System development**	30	39	1.21E-03
Multicellular organismal process	52	70	1.22E-03

The 540 probe sets of significantly upregulated or downregulated probes identified in RD cells expressing pB-PAX3(+/−Q)-FOXO1-ER in the HG-U133+ v.2.0 platform were analyzed using DAVID. The most significant 20 functional groups for either upregulated or downregulate genes are listed. DAVID, Database for Annotation, Visualization and Integrated Discovery; pB, pBabe.

**Table III:** Validation of the expression of 4 target genes (GREM1, DAPK1, MYOD1 and HEY1).

	Fold-change				
	
	Microarray		qRT-PCR		
		
	Tumors	Inducible cells	Tumors	P-values (tumors)	Inducible cells
Downregulated genes					
HEY1	6.21	1.72	4.83	0.0000	2.14
Upregulated genes					
GREM1	5.34	2.13	6.82	0.0000	1.76
DAPK1	2.51	2.40	4.26	0.0000	2.17
MYOD1	2.59	2.28	1.86	0.0033	2.11

The expression of genes was determined by qRT-PCR in independent tumors (20 ERMS; 17 P3F-positive ARMS) and RD ERMS cells transduced with inducible PAX3(+/−Q)-FOXO1-ER in pB. For microarray, 31 tumors (15 ERMS; 16 P3F-positive ARMS) and 12 inducible cell culture samples (6 Tmf-treated, 6 untreated) were analyzed. The fold-change difference was calculated by dividing the expression levels of P3F-positive categories of tumors (ARMS) and inducible cells with those of P3F-negative categories of tumors (ERMS) and inducible cells. GREM1, gremlin1, cysteine knot superfamily 1, BMP antagonist 1; DAPK1, death-associated protein kinase 1; MYOD1, myogenic differentiation 1; HEY1, hairy/enhancer-of-split related with YRPW motif 1; qRT-PCR, quantitative reverse-transcription PCR; ERMS, embryonal rhabdomyosarcoma; P3F, PAX3-FOXO1; ARMS, alveolar rhabdomyosarcoma; pB, pBabe; Tmf, 4-hydroxytamoxifen.

**Table IV:** Comparison of the potential target genes of PAX3-FOXO1 in three studies (13,16, present study).

6 common genes in all three studies [Davicioni *et al*(13), Mercado *et al*(16) and present study]	12 common genes in present study and Mercado *et al*(16)	11 common genes in present study and Davicioni *et al*(13)	16 common genes in Davicioni *et al*(13) and Mercado *et al*(16)
KCNN3	DAPK1	ABAT	DCX
MCAM	DKFZp762M127	IL4R	DUSP4 (↓)
MYOD1	GREM1	INPP1	GADD45A
TCF7L2	HEY1 (↓)	KCNN3	IGFBP3 (↓)
TM4SF10	KCNN3	MCAM	KCNN3
ZFP36L2 (↓)	MCAM	MYOD1	MARCH3
	MYOD1	NRCAM	MCAM
	NCOA1	SKP2	MEG3
	QDPR	TCF7L2	MET
	TCF7L2	TM4SF10	MYCN
	TM4SF10	ZFP36L2 (↓)	MYOD1
	ZFP36L2 (↓)		NEBL
			PRKAR2B
			TCF7L2
			TM4SF10
			ZFP36L2 (↓)

The potential target genes of P3F were identified by microarray analysis in both primary RMS tumors and cells transduced with constitutive PAX3/PAX7-FOXO1 (13) or inducible P3F-ER (16, present study) constructs. Unless noted as (↓) for downregulated genes, all other genes were upregulated genes.

## Abbreviations

RMS: rhabdomyosarcoma
Q: glutamine
ARMS: alveolar rhabdomyosarcoma
ERMS: embryonal rhabdomyosarcoma
ER: estrogen receptor
pK: pK1
P3F: PAX3-FOXO1
P3F-ER: PAX3-FOXO1-ER
Tmf: 4-hydroxytamoxifen
